# Cyclic Peptide [R_4_W_4_] in Improving the Ability of First-Line Antibiotics to Inhibit *Mycobacterium tuberculosis* Inside *in vitro* Human Granulomas

**DOI:** 10.3389/fimmu.2020.01677

**Published:** 2020-08-13

**Authors:** Joshua Hernandez, David Ashley, Ruoqiong Cao, Rachel Abrahem, Timothy Nguyen, Kimberly To, Aram Yegiazaryan, Ajayi Akinwale David, Rakesh Kumar Tiwari, Vishwanath Venketaraman

**Affiliations:** ^1^Graduate College of Biomedical Sciences, Western University of Health Sciences, Pomona, CA, United States; ^2^College of Osteopathic Medicine of the Pacific, Western University of Health Sciences, Pomona, CA, United States; ^3^Department of Basic Medical Sciences, College of Osteopathic Medicine of the Pacific, Western University of Health Sciences, Pomona, CA, United States; ^4^Department of Biomedical and Pharmaceutical Sciences, Center for Targeted Drug Delivery, Chapman University School of Pharmacy, Harry and Diane Rinker Health Science Campus, Irvine, CA, United States

**Keywords:** cyclic peptide, tuberculosis, host–bacteria interaction, cytokine, antimicrobial peptides

## Abstract

Tuberculosis (TB) is currently one of the leading causes of global mortality. Medical non-compliance due to the length of the treatment and antibiotic side effects has led to the emergence of multidrug-resistant (MDR) strains of *Mycobacterium tuberculosis* (*M. tb*) that are difficult to treat. A current therapeutic strategy attempting to circumvent this issue aims to enhance drug delivery to reduce the duration of the antibiotic regimen or dosage of first-line antibiotics. One such agent that may help is cyclic peptide [R_4_W_4_], as it has been shown to have antibacterial properties (in combination with tetracycline) against methicillin-resistant *Staphylococcus aureus* (MRSA) in the past. The objective of this study is to test cyclic peptide [R_4_W_4_] both alone and in combination with current first-line antibiotics (either isoniazid or pyrazinamide) to study the effects of inhibition of *M. tb* inside *in vitro* human granulomas. Results from our studies indicate that [R_4_W_4_] is efficacious in controlling *M. tb* infection in the granulomas and has enhanced inhibitory effects in the presence of first-line antibiotics.

## Introduction

*Mycobacterium tuberculosis* (*M. tb*) is the etiological agent that is responsible for causing tuberculosis (TB). According to the World Health Organization (WHO), it is estimated that around 9 million people are suffering from active TB disease with an approximate global mortality of 1.43 million people annually (1, 2). Furthermore, about a quarter of the world's population is affected with latent TB (1, 2).

Current treatment for drug-sensitive TB includes first-line antibiotics, which are administered for approximately 6–9 months (3). Although treatment may be effective, problems with drug toxicity can lead to severe side effects, interfering with patients' compliance with medical treatment. Non-compliance to treatment often leads to the emergence of multidrug-resistant (MDR) strains of *M. tb* and the development of MDR-TB, which is difficult to treat, highlighting the need for new therapeutic strategies.

Novel therapeutic approaches that can achieve complete cure along with reduced duration of treatment and dosage of first-line anti-TB drugs are highly warranted. These strategies may be crucial in alleviating drug side effects and in addressing patient non-compliance.

**Figure 1:**
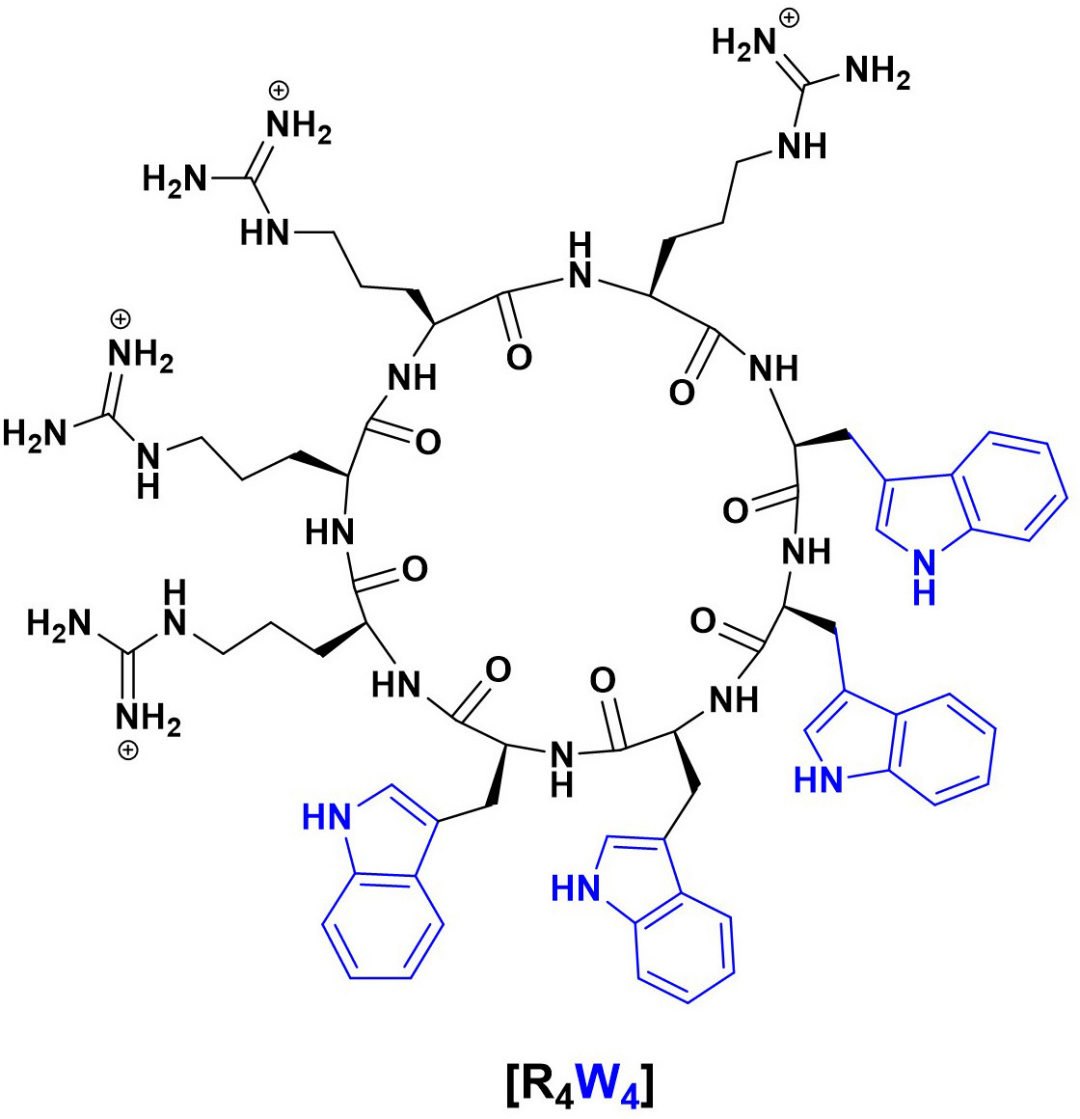
Chemical structure of cyclic peptide [R_4_W_4_].

Antimicrobial peptides (AMPs) are one such alternative therapeutics against antibiotic-resistant pathogens since they may act as effectors and regulators of the immune system as well as inhibitors of bacterial cell growth (4). Additionally, there are cell-penetrating peptides (CPPs) that share amphiphilicity and cationic structural properties with antimicrobial peptides. Cell-penetrating peptides are particularly interesting since they may help deliver other drugs intracellularly due to their ability to move across the eukaryotic cell membrane. In the grand scheme, it is thought that the correlation between antimicrobial activity and cell-penetrating property may be due to the interaction between positively charged amphiphilic peptides and bacterial membranes that have negatively charged components (4). One such antimicrobial peptide is cyclic peptide [R_4_W_4_], which has a cyclic structure consisting of four arginine and four tryptophan residues (Figure 1), enabling it to interact with cell membranes and deliver cargo. We, therefore, tested the effects of [R_4_W_4_] both alone and in combination with the first-line antibiotics in restricting the growth of *M. tb* inside the *in vitro* generated human granulomas.

Our study findings indicate that [R_4_W_4_] causes a significant decrease in the viability of *M. tb* and works effectively with first-line antibiotics such as isoniazid (INH) and pyrazinamide (PZA) by modulating the levels of cytokine release, oxidative stress, and autophagy.

## Materials and Methods

### Human Subjects and Whole Blood Collection

In this study, we recruited eight healthy human subjects aged between 18 and 65 years irrespective of other demographic characteristics. Subjects were excluded if they self-reported ever having a positive purified protein derivative (PPD) skin test, a *Bacille Calmette-Guerin* (BCG) vaccination, a history of excessive alcohol intake, and/or chronic Hepatitis B or C infection(s). Informed consent was obtained from each subject prior to the initiation of any research procedures. After obtaining written informed consent, ~35 ml of whole blood was obtained from each subject using Acid Citrate Dextrose tubes by research nursing staff at the WesternU Health Patient Care Center. All studies were approved by the Institutional Review Board (IRB) and the Institutional Biosafety Committee (IBC) of the Western University of Health Sciences. All study participants were above the legal age of consent at the time of participation, and written informed consent was obtained from all volunteers prior to participation in the study.

### Isolation of Peripheral Blood Mononuclear Cells

Whole blood samples were distributed between two 50-ml conical tubes containing Ficoll-Histopaque (Sigma, St. Louis, MO, USA) at a 1:1 ratio. Following centrifugation (1,800 rpm for 30 min), peripheral blood mononuclear cells (PBMCs) were collected and washed twice with sterilized 1 × phosphate buffer saline (PBS) (Sigma, St. Louis, MO, USA). PBMCs were suspended in RPMI with 5% human AB serum (Sigma, St. Louis, MO, USA). Cell counts were achieved using Trypan Blue exclusion staining and a hemocytometer.

### Infection, Treatment, and Termination of PBMC-Derived Granulomas

PBMCs (6 × 10^5^) with verified counts were infected with *M. tb* (Erdman strain) using a multiplicity of infection ratio of 1:10 (*M. tb*: PBMCs) and distributed into sterile cell culture 24-well plates (Corning, Corning, NY, USA). Two wells per category contained cover glasses that were used to collect *in vitro* granulomas for staining procedures. Treatments were applied one time in quadruplicate using the following categories: Control (sham treatment), INH, PZA, [R_4_W_4_] (4 μg/ml), [R_4_W_4_] (8 μg/ml), INH and [R_4_W_4_] (4 μg/ml), INH and [R_4_W_4_] (8 μg/ml), PZA and [R_4_W_4_] (4 μg/ml), and PZA and [R_4_W_4_] (8 μg/ml). All treatments that included INH and PZA used minimum inhibitory concentrations (0.125 μg/ml and 50 μg/ml, respectively). Treated infected PBMCs were cultured for 8 days at 37°C in the presence of 5% CO_2_ for granuloma formation. At the conclusion of the 8-day incubation, each well was terminated. Supernatants of each well were collected and aliquoted into sterile Eppendorf tubes by treatment categories. Sterile cold 1 × PBS was added into each experimental well without cover glasses to lyse the *in vitro* granulomas. To further dislodge the *in vitro* granulomas, each well was scraped using a sterile micropipette. Lysates from each category were then collected and aliquoted into sterile Eppendorf tubes by treatment category. Wells with cover glasses were treated with 4% paraformaldehyde (PFA) for 1 h at room temperature to affix *in vitro* granulomas to their respective cover glasses for imaging analyses. PFA-treated wells were also washed three times with PBS to remove any cellular debris that could impede imaging analyses.

### Quantification of Intracellular *M. tb* Survival

In order to quantify the intracellular survival of *M. tb* in these treated *in vitro* granulomas, plates containing Middlebrook 7H11 agar media (Hi Media, Santa Maria, CA, USA) supplemented with albumin-dextrose-catalase (ADC) (GEMINI, Calabasas, CA) were inoculated with previously collected supernatants and lysates. Plates were then incubated at 37°C for a minimum of 4 weeks. Plates were then read, and colony-forming units (CFUs) were counted.

### Cytokine Measurements

Cytokine analyses were conducted to quantify the produced levels of TNF-α, IFN-γ, and IL-10 in collected supernatant samples. The sandwich enzyme-linked immunosorbent assay (ELISA) was used. Assays and analyses were conducted per the assay manufacturer's protocol (Invitrogen, Carlsbad, CA, USA).

### Imaging of *in vitro* Granulomas

Cover glasses with fixed *in vitro* granulomas were permeabilized with Triton-X for 2 min and stained overnight with FITC-conjugated CellROX and with antibodies against LC3B conjugated with PE. Cover glasses were washed with PBS and mounted on clean glass slides with mounting media containing 4′,6-diamidino-2-phenylindole (DAPI). Slides were observed under the fluorescent microscope. Fluorescent images were captured, and the fluorescent intensity was quantified using the ImageJ software.

### Statistical Analysis

All data analyses for this study were conducted using GraphPad Prism 8.0. (version 8, GraphPad, San Diego, CA, USA). A one-way ANOVA (analysis of variance) with Tukey corrections was performed for datasets greater than two groups. Reported values are in means with each respective category. Data represent ±SE from experiments performed in eight different individuals. Analyses with a *p* < 0.05 was considered statistically significant.

## Results and Discussion

Antimicrobial peptides are promising candidates that can be used as adjunctive therapy for TB. The structural and functional qualities of the synthetic AMP, [R_4_W_4_], led us to test its efficacy against *M. tb*., which has a thick peptidoglycan layer along with other lipid layers on the cell wall. [R_4_W_4_] has already been shown to have potent antibacterial activity against pathogenic gram-positive bacteria such as methicillin-resistant *Staphylococcus aureus* (MRSA) with an inhibitory concentration (MIC) of 2.67 μg/ml (1.95 μM). In addition, a follow-up 24-h study showed bactericidal activity against MRSA with the combination of two times the MIC of [R_4_W_4_] and four times the MIC of tetracycline (0.5 μg/ml) (4). Cytotoxicity studies of [R_4_W_4_] have been reported using MTS proliferation assay against two cancer cell lines (human ovarian adenocarcinoma SK-OV-3 and human leukemia CCRF-CEM) and one normal human embryonic kidney HEK 293T cell line at 24-h incubation. The cyclic peptide [R_4_W_4_] demonstrated more than 84% cell viability at a concentration of 20.5 μg/ml in both cancer and normal cell lines (4). Furthermore, [R_4_W_4_] showed minimal hemolytic activity by showing <10% hemolysis of normal red blood cells at the concentration of 128 μg/ml. Therefore, these studies demonstrate negligible or minimum cytotoxicity of [R_4_W_4_] up to 20.5 μg/ml to the normal and cancerous cell lines. A fluorescent-labeled [R_4_W_4_] peptide was synthesized and reported to disperse into the nucleus and cytoplasm. This demonstrated the transporter property of the [R_4_W_4_] peptide. Furthermore, it has been reported that cyclic peptides have higher stability against proteases and found resistant toward proteolytic degradation as compared to linear peptides (5). Cyclic peptides have demonstrated stability under disease condition, and some of them are successfully used as drugs, including vancomycin, daptomycin, polymyxin B, colistin, caspofungin, and cyclosporine (6–8). A series of cyclic WR peptides has been reported to be stable against serum and found to have molecular transporter properties (9, 10). Therefore, [R_4_W_4_] will be stable against proteases due to very similar amino acid composition and cyclic nature. As [R_4_W_4_] has shown bactericidal activity (alongside tetracycline) against MRSA (4), it is an attractive candidate that can be tested along with first-line anti-TB drugs against *M. tb*.

Two of the first-line antibiotics used to treat active TB are INH and PZA, both of which have MICs of 0.125 and 50 μg/ml, respectively, against *M. tb*. In our past studies, one-time treatment of *in vitro M tb*-infected granulomas (or immune cell clusters) with lone INH and PZA at MIC did not result in complete clearance of *M. tb*. (11). We, therefore, tested the effects of [R_4_W_4_] both alone and in combination with either INH or PZA against *M. tb* in the *in vitro* granulomas generated from PBMCs isolated from healthy subjects.

**Figure 2:**
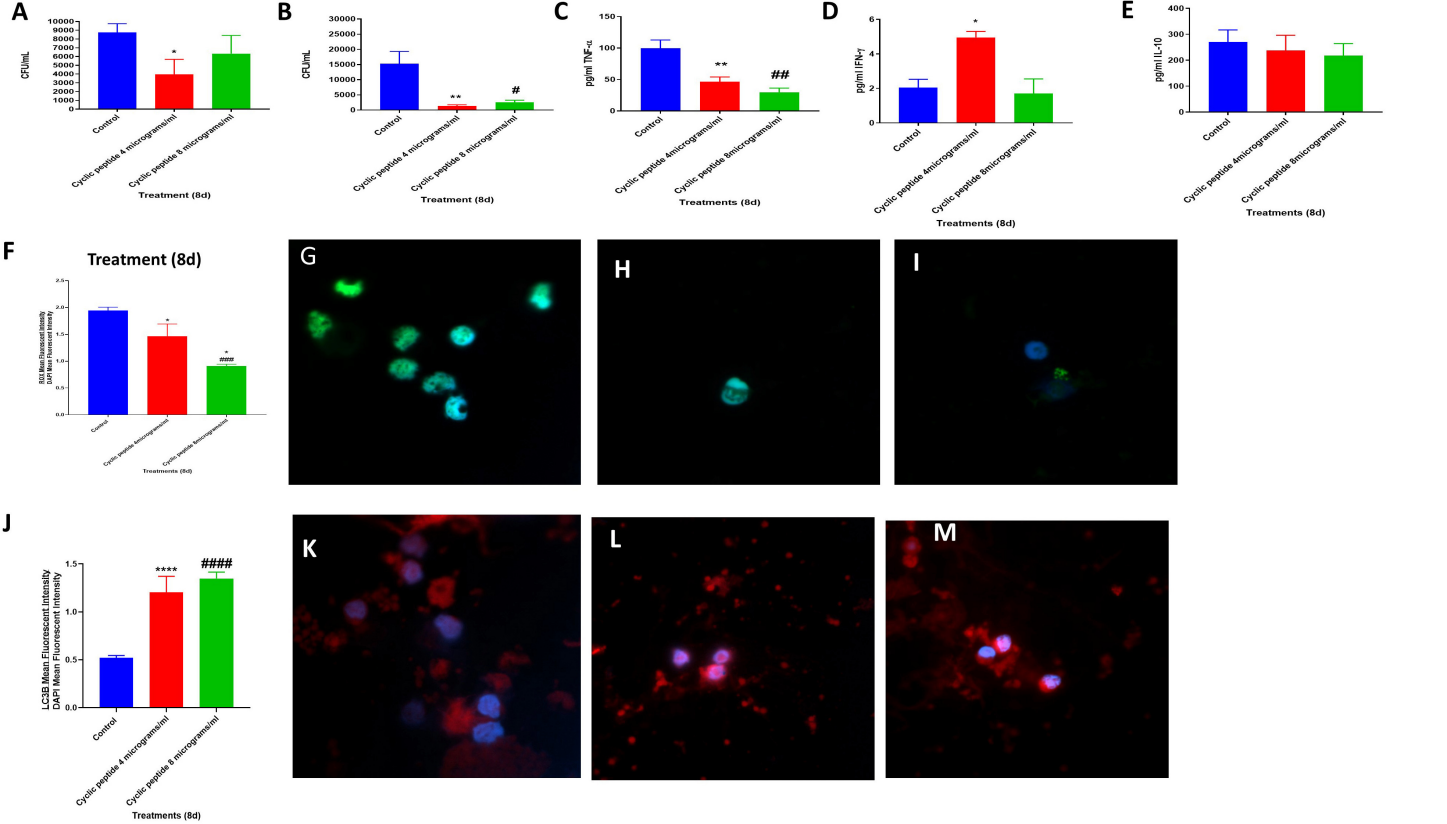
Efficacy of [R_4_W_4_] against *M. tb*. Sensitivity Erdmann strain of *M. tb* to the cyclic peptide [R_4_W_4_] added at 4 and 8 μg/ml concentrations. *P < 0.05 when comparing CFUs at the 8-day time point of 4 μg/ml [R_4_W_4_]-treated Erdman strain to untreated control category **(A)**. Effects of [R_4_W_4_] in altering the viability in *M. tb* in the *in-vitro* granulomas **(B)**. Effects of [R_4_W_4_] in altering the production of TNF-α **(C)**. Effects of [R_4_W_4_] in altering the levels of IFN-γ **(D)**. Effects of [R_4_W_4_] in altering the production of IL-10 **(E)**. Effects of [R_4_W_4_] on reactive oxygen species production **(F)**. Mean fluorescent intensity of CellROX-stained (green fluorescent) control granulomas **(G)**, [R_4_W_4_] (4 μg/ml)-treated **(H)**, and [R_4_W_4_] (8 μg/ml)-treated granulomas **(I)**. Effects of [R_4_W_4_] on LC3B expression **(J)**. Mean fluorescent intensities of LC3B-stained (PEt)control granulomas **(K)**, [R_4_W_4_] (4 μg/ml)-treated **(L)**, and [R_4_W_4_] (8 μg/ml)-treated granulomas **(M)**. Anti-LC3B conjugated with PE and CellROX conjugated with FITC were applied on the same specimen. Fluorescent images were captured for each marker either from the same field or from a different representative field. Reported values are in means with each respective category. Data represent ± SE from experiments performed from eight different individuals. Analysis of figures utilized a one-way ANOVA with Tukey test. The *p* value above 0.05 is not considered significant, one symbol (* or #) denotes significant difference below 0.05, two symbols (** or ##) denote significant difference below 0.005, three symbols (*** or ###) denote significant difference below 0.0005, and four symbols (**** or ####) denote significant difference below 0.0001. An asterisk (*) indicates comparison to the direct previous category. A hash mark (#) indicates comparison between one category and a previous category that is one column before it. A dollar sign ($) indicates comparison between one category and a previous category that is two columns before it.

We first tested the direct antimycobacterial effects of [R_4_W_4_] added at 4 and 8 μg/ml concentrations to static cultures of Erdman strain of *M. tb*. Static cultures of *M. tb* were grown in 7H9 media in the presence and absence of [R_4_W_4_] (added at 4 μg/ml and 8 μg/ml) and the growth of *M. tb* was monitored for 8 days (Figure 2A). The selection of the two concentrations (4 μg/ml and 8 μg/ml) of [R_4_W_4_] was based on the rationale of reported MIC values of peptide [R_4_W_4_] against MRSA (4, 12, 13). The bacterial suspension was plated on 7H11 media and incubated for 4 weeks for colony formation. Compared to the untreated control group, *M. tb* survival (demonstrated by CFU counts) in 7H9 was significantly reduced by 2.2-fold in the group treated with [R_4_W_4_] at 4 μg/ml concentration (*p* = 0.0223) (Figure 2A). There was also reduced *M. tb* survival in the group treated with [R_4_W_4_] at 8 μg/ml, but this was not a statistically significant finding (*p* = 0.1709) (Figure 2A).

We then determined the viability of *M. tb* inside the *in vitro* granulomas treated with [R_4_W_4_] at 4 and 8 μg/ml. The sham control group of human *M. tb*-infected granulomas was compared against *in vitro* granulomas treated with [R_4_W_4_] at 4 and 8 μg/ml, respectively. Compared to the control group, intracellular *M. tb* survival was significantly reduced in both groups treated with [R_4_W_4_] at 4 μg/ml (*p* = 0.0348) and 8 μg/ml concentrations (*p* = 0.0423) (Figure 2B). In comparison to the untreated granulomas, the fold reduction in the CFUs was 10.86 and 5.95 for granulomas treated with [R_4_W_4_] at 4 and 8 μg/ml, respectively. However, there was no significant difference in the intracellular *M. tb* survival between the groups treated with 4 and 8 μg/ml (*p* = 0.2719) (Figure 2B). These findings indicate that the treatment of *in vitro* granulomas with R_4_W_4_ caused a reduction in the *M. tb* burden (Figure 2B).

To understand the effects of [R_4_W_4_] in altering the underlying immune effector mechanisms against *M. tb* infection, we first measured the levels of several related cytokines that cause the activation (TNF-α and IFN-γ) and inhibition (IL-10) of immune effector mechanisms. TNF-α expression was significantly downregulated by [R_4_W_4_] at both 4 μg/ml (*p* = 0.036) and 8 μg/ml concentrations (*p* = 0.0003) compared to sham-treated control categories (Figure 2C). In comparison to the untreated granulomas, the fold reduction in the levels of TNF-α was 2 and 3.3 for granulomas treated with [R_4_W_4_] at 4 and 8 μg/ml, respectively. TNF-α, a pro-inflammatory cytokine, activates the effector immune functions and causes recruitment of immune cells to form a solid and stable granuloma to contain *M. tb* infection (14–18). Excess TNF-α can cause cell death by necrosis, leading to inflammation (19, 20). Our findings indicate a positive correlation between diminished numbers of *M. tb* in [R_4_W_4_]-treated granulomas with a corresponding decrease in the levels of TNF-α explaining the immunomodulatory effects of [R_4_W_4_].

IFN-γ is crucial in both innate and adaptive immunity, acting as a macrophage activation factor and mediating MHC molecule expression (21). There was a 2.5-fold increase in IFN-γ production in the supernatants from *in vitro* granulomas treated with 4 μg/ml concentration of [R_4_W_4_] (*p* = 0.0161) compared to sham control (Figure 2D). In our previously published study (18), we demonstrated that IFN-γ increase was associated with enhanced phagosome acidification and improved killing of *M. tb* in the *in vitro* granulomas. Therefore, higher amounts of the IFN-γ release may therefore serve as an additional effector mechanism by which *in vitro* granulomas treated with 4 μg/ml of [R_4_W_4_] control *M. tb* infection.

IL-10 is an immune-suppressive cytokine that can inhibit phagosome–lysosome fusion and dampen other effector responses against *M. tb* infection (11, 22–24). We observed a notable decrease in the levels of IL-10 in [R_4_W_4_]**-**treated granulomas at both concentrations (4 and 8 μg/ml). These findings indicate that [R_4_W_4_] supplementation augments immune responses against *M. tb* infection by downregulating the levels of IL-10 (Figure 2E) and by increasing the levels of IFN-γ (Figure 2D).

It is well-known that oxidative stress generated during *M. tb* infection can contribute to both protection and pathogenesis (25). *M. tb* infection triggers the release of pro-inflammatory cytokines and reactive oxygen species (ROS) leading to oxidative stress. It is important to point out that the survival of *M. tb* is dependent on the extent of ROS produced by the host immune cells. Exacerbation in the levels of ROS can damage the immune cells causing diminishment in the effector responses against *M. tb* infection (25, 26). CellROX staining was performed to determine the extent of oxidative stress in sham control and [R_4_W_4_]-treated granulomas (Figure 2F). In comparison to the control group, there was a significant decrease in the intensity of CellROX staining in the [R_4_W_4_]-treated granulomas (Figures 2F–I). In comparison to the untreated granulomas, the fold reduction in the intensity of CellROX staining was 1.3 and 2.1 for granulomas treated with [R_4_W_4_] at 4 and 8 μg/ml, respectively. This significant reduction in the mean fluorescence intensity implies attenuation of oxidative stress levels. These data further illustrate that reducing the oxidative stress restores favorable immune responses against *M. tb* infection. TNF-α plays a central role in triggering the inflammatory response via ROS production (18, 27, 28). Additionally, ROS can induce TNF-α production (18, 27). Our study findings therefore confirm this direct link between TNF-α levels and oxidative stress and the effects of [R_4_W_4_] in downregulating the levels of both.

Autophagy is a self-degradative process, which plays a critical role in promoting cellular senescence, antigen presentation on the cell surface, and eliminating intracellular waste aggregates and damaged organelles, thereby preventing necrosis (29–31). It has been reported that autophagy functionally benefits some diseases such as cancer, cardiomyopathy, diabetes, liver disease, and infections (29–31). LC3B is a mammalian homolog of the yeast ATG8 protein, which is a ubiquitin-like protein related to autophagosomal membranes (32, 33). Therefore, LC3B is an important and direct marker used for detecting autophagy levels (32, 33). The LC3B protein in the granulomas were stained by an anti-LC3B antibody tagged with PE, a red fluorescent dye. Increased LC3B expression is indicative of increased autophagy. We observed that the mean fluorescence intensity of LC3B was significantly elevated in the [R_4_W_4_]**-**treated granulomas at both concentrations compared to the untreated granulomas (Figures 2J–M). In comparison to the untreated granulomas, the fold increase in the expression levels of LC3B was 2.4 and 2.6 for granulomas treated with [R_4_W_4_] at 4 and 8 μg/ml, respectively. Our results specify that autophagy may be one of the immune effector mechanisms by which [R_4_W_4_]-treated *in vitro* granulomas control *M. tb* infection.

**Figure 3:**
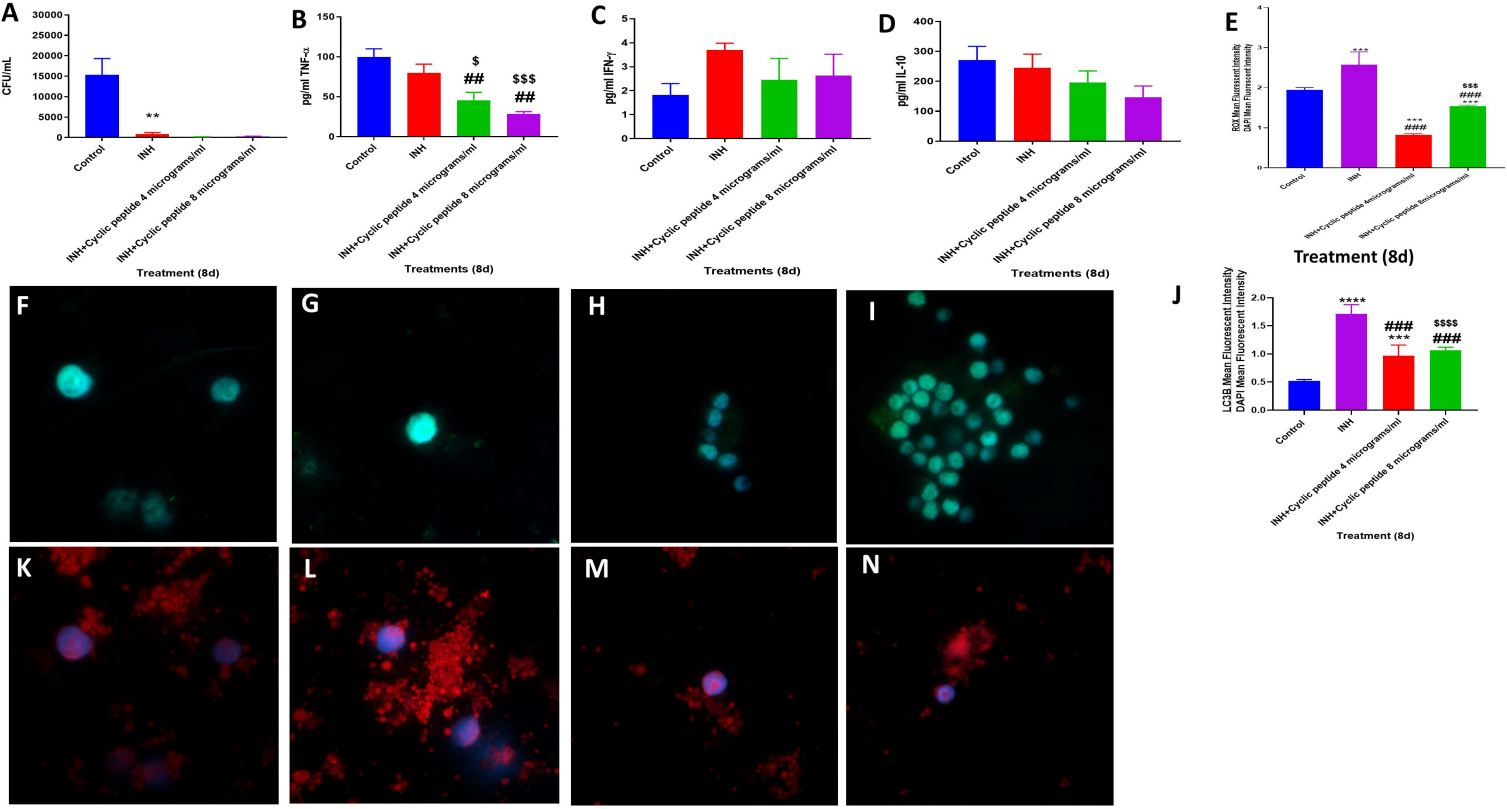
Efficacy of INH and INH + [R_4_W_4_] against *M. tb*. Effects of INH and INH + [R_4_W_4_] (added at 4 and 8 μg/ml concentrations) in altering the viability in *M. tb* in the *in vitro* granulomas **(A)**. Effects of INH and INH + [R_4_W_4_] (added at 4 and 8 μg/ml concentrations) in interfering with the production of TNF-α **(B)**. Effects of INH and INH + [R_4_W_4_] (added at 4 and 8 μg/ml concentrations) in interfering with the production of IFN-γ **(C)**. Effects of INH and INH + [R_4_W_4_] (added at 4 and 8 μg/ml concentrations) in interfering with the production of IL-10 **(D)**. Effects of INH and INH + [R_4_W_4_] (added at 4 and 8 μg/ml concentrations) on reactive oxygen species production **(E)**. Mean fluorescent intensity of CellROX-stained (green fluorescent) control granulomas **(F)**, INH-treated **(G)**, INH + [R_4_W_4_] (4 μg/ml)-treated **(H)**, and INH + [R_4_W_4_] (8 μg/ml)-treated granulomas **(I)**. Effects of INH and INH+ [R_4_W_4_] (added at 4 and 8 μg/ml concentrations) on LC3B expression **(J)**. Mean fluorescent intensities of LC3B-stained (red fluorescent) control granulomas **(K)**, INH-treated **(L)**, INH + [R_4_W_4_] (4 μg/ml)-treated **(M)**, and INH + [R_4_W_4_] (8 μg/ml)-treated granulomas **(N)**. Anti-LC3B conjugated with PE (red) and CellROX conjugated with FITC (green) were applied on the same specimen. Fluorescent images were captured for each marker either from the same field or from a different representative field. Reported values are in means with each respective category. Data represents ± SE from experiments performed from 8 different individuals. Analysis of figures utilized a one-way ANOVA with Tukey test. The *p* value above 0.05 is not considered significant, one symbol (*, #, or $) denotes significant difference below 0.05, two symbols (**, ##, or $$) denote significant difference below 0.005, three symbols (***, ###, or $$$) denote significant difference below 0.0005, and four symbols (****, ####, or $$$$) denote significant difference below 0.0001. An asterisk (*) indicates comparison to the direct previous category. A hash mark (#) indicates comparison between one category and a previous category that is one column before it. A dollar sign ($) indicates comparison between one category and a previous category that is two columns before it.

We also tested the effects of [R_4_W_4_] and first-line antibiotics (INH or PZA) in reducing the burden of *M. tb* in the *in vitro* granuloma*s*. Infected PBMCs were treated with [R_4_W_4_] at 4 and 8 μg/ml concentrations and cultured in the presence and absence of INH or PZA at their respective MIC. Consistent with our previous observations, there was a significant and 18-fold reduction in viability of *M. tb* in INH-treated granulomas (*p* = 0.0314). Treatment with INH in conjunction with [R_4_W_4_] at either 4 μg/ml (*p* = 0.0287) or 8 μg/ml (*p* = 0.0292) concentration resulted in a further reduction in the viability of *M. tb* when compared with the control category (Figure 3A). In comparison to the untreated granulomas, the fold reduction in the viability of *M. tb* was 104 and 57 in granulomas treated with INH + [R_4_W_4_] at 4 μg/ml and INH + [R_4_W_4_] 8 μg/ml, respectively. Although these findings were statistically significant, there were no significant statistical findings when comparing the INH category with INH treatment in conjunction with [R_4_W_4_] at 4 μg/ml (*p* = 0.2787) and 8 μg/ml (*p* = 0.3803) concentrations (Figure 3A).

The reduction in the viability of *M. tb* in INH + [R_4_W_4_]-treated granulomas was accompanied by a significant diminishment in the levels of TNF-α (*p* = 0.0113) along with a notable decrease in the levels of IL-10 (*p* = 0.1336) in the granuloma supernatants from INH + [R_4_W_4_] treatment categories when compared to untreated and INH-alone treated groups (Figures 3B,D). INH + [R_4_W_4_] treatment did not result in any significant changes in the production of IFN-γ (Figure 3C). Furthermore, treatment with INH + [R_4_W_4_] resulted in a significant decrease in the intensity of CellROX when compared to INH alone and control categories (Figures 3E–I). INH + [R_4_W_4_] treatment also resulted in a significant decrease in the intensity of LC3B staining when compared to the INH-alone treatment group. However, when compared to the control category, INH + [R_4_W_4_] treatment resulted in a statistically significant 2-fold increase in the intensity of LC3B staining (Figures 3J–N). Our results demonstrate that INH, when given in conjunction with [R_4_W_4_], decreases *M. tb* burden and downregulates oxidative stress and production of TNF-α and IL-10, when compared to lone INH treatment and control. Although the combination of INH + [R_4_W_4_] did not increase the expression of LC3B when compared to treatment with INH alone, INH + [R_4_W_4_] treatment downregulated the intensity of CellROX staining and diminished the production of TNF-α and IL-10. Although TNF-α plays a central role in the formation and maintenance of granulomas, overexpression of TNF-α has also been linked to many inflammatory and autoimmune diseases (14–20). IL-10, an immunosuppressive cytokine, can downregulate the effector functions of macrophage against *M. tb* infection (11, 22–24, 34). Therefore, combination of [R_4_W_4_] with INH can downregulate oxidative stress and production of TNF-α and IL-10, which in turn can favor improved control of *M. tb* infection when compared to treatment with INH alone.

**Figure 4:**
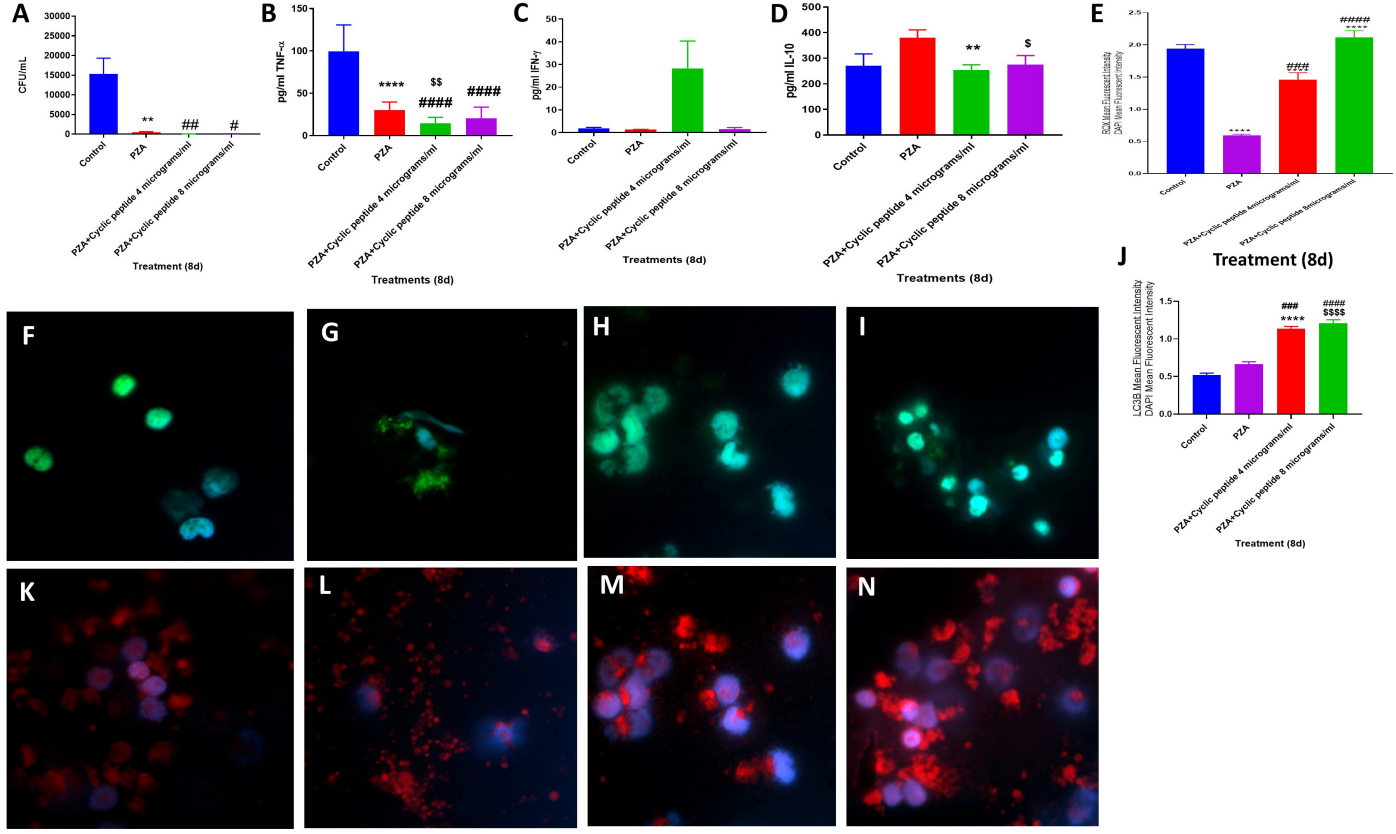
Efficacy of PZA and PZA + [R_4_W_4_] against *M. tb*. Effects of PZA and PZA + [R_4_W_4_] (added at 4 and 8 μg/ml concentrations) in altering the viability in *M. tb* in the *in vitro* granulomas **(A)**. Effects of PZA and PZA + [R_4_W_4_] (added at 4 and 8 μg/ml concentrations) in interfering with the production of TNF-α **(B)**. Effects of PZA and PZA + [R_4_W_4_] (added at 4 and 8 μg/ml concentrations) in interfering with the production of IFN-γ **(C)**. Effects of PZA and PZA + [R_4_W_4_] (added at 4 and 8 μg/ml concentrations) in interfering with the production of IL-10 **(D)**. Effects of PZA and PZA + [R_4_W_4_] (added at 4 and 8 μg/ml concentrations) on reactive oxygen species production **(E)**. Mean fluorescent intensity of CellROX-stained (green fluorescent) control granulomas **(F)**, PZA-treated **(G)**, PZA + [R_4_W_4_] (4 μg/ml)-treated **(H)**, and PZA + [R_4_W_4_] (8 μg/ml)-treated granulomas **(I)**. Effects of PZA and PZA+ [R_4_W_4_] (added at 4 and 8 μg/ml concentrations) on LC3B expression **(J)**. Mean fluorescent intensities of LC3B-stained (red fluorescent) control granulomas **(K)**, PZA-treated **(L)**, PZA + [R_4_W_4_] (4 μg/ml)-treated **(M)**, and PZA + [R_4_W_4_] (8 μg/ml)-treated granulomas **(N)**. Anti-LC3B conjugated with PE (red) and CellROX conjugated with FITC (green) were applied on the same specimen. Fluorescent images were captured for each marker either from the same field or from a different representative field. Reported values are in means with each respective category. Data represents ± SE from experiments performed from 8 different individuals. Analysis of figures utilized a one-way ANOVA with Tukey test. The *p* value above 0.05 is not considered significant, one symbol (*, #, or $) denotes significant difference below 0.05, two symbols (**, ##, or $$) denote significant difference below 0.005, three symbols (***, ###, or $$$) denote significant difference below 0.0005, and four symbols (****, ####, or $$$$) denote significant difference below 0.0001. An asterisk (*) indicates comparison to the direct previous category. A hash mark (#) indicates comparison between one category and a previous category that is one column before it. A dollar sign ($) indicates comparison between one category and a previous category that is two columns before it.

When compared to the untreated control category, PZA treatment also resulted in a statistically significant and 30-fold reduction in the viability of *M. tb* in the granulomas (Figure 4A) (*p* = 0.0302). A further decrease in the viability of *M. tb* was observed with PZA + [R_4_W_4_] treatments. The fold reduction in the viability of *M. tb* was 511 and 136 in granulomas treated with PZA + [R_4_W_4_] at 4 μg/ml and PZA + [R_4_W_4_] 8 μg/ml, respectively, when compared to untreated granuloma. The levels of TNF-α, IL-10, and IFN-γ were measured in the granuloma supernatants from control, PZA-treated, and PZA + [R_4_W_4_]-treated groups (Figures 4B–D). There was a statistically significant and 3.3-fold reduction in the levels of TNF-α in the PZA-treated group when compared to the control (Figure 4B). PZA + [R_4_W_4_] treatment resulted in a further significant decrease in the production of TNF-α when compared to the PZA-alone category and control groups. PZA + [R_4_W_4_] treatment also resulted in a significant decrease in the production of IL-10 (Figure 4D).

Although not significant, PZA + [R_4_W_4_] (4 μg/ml) treatment resulted in an increase in the levels of IFN-γ (Figure 4C). PZA treatment resulted in attenuation in the fluorescence intensity of CellROX. However, the granulomas treated with PZA + [R_4_W_4_] showed a significant increase in the intensity of CellROX staining compared to the PZA-alone group (Figures 4E–I). There was increased intensity of LC3B staining in PZA-treated granulomas compared to the untreated control group. PZA + [R_4_W_4_] treatment caused a significant increase in the intensity of LC3B staining compared to the control and PZA-alone group. In comparison to the untreated granulomas, the fold increase in the intensity of LC3B staining was 2 and 2.4 in granulomas treated with PZA + [R_4_W_4_] at 4 μg/ml and PZA + [R_4_W_4_] at 8 μg/ml, respectively (Figures 4J–N). Our results indicate that when compared to the PZA-alone category, treatment with PZA + [R_4_W_4_] caused increased production of IFN-γ, decreased levels of IL-10, increased autophagy, and improved control of *M. tb* infection. These findings further support the previous findings that decreased IL-10 along with increased IFN-γ will favor effective control of *M. tb* infection (21).

Our study findings demonstrate that [R_4_W_4_] elicits direct antimycobacterial ability against virulent Erdman strain of *M. tb* at 4 μg/ml. Treatment of *in vitro* granulomas with [R_4_W_4_] both alone and in combination with first-line antibiotics such as INH and PZA at MIC concentration resulted in a further decrease in the viability of *M. tb*. The addition of [R_4_W_4_] to granulomas both in the presence and absence of PZA led to a reduction in the levels of TNF-α and IL-10, and elevation in the levels of autophagy. Our study findings, therefore, indicate that [R_4_W_4_] causes a significant decrease in the viability of *M. tb* and works in conjunction with first-line antibiotics by modulating the levels of cytokine release and autophagy. In conclusion, we believe that [R_4_W_4_], along with anti-TB treatment, would not only attenuate the side effects of antibiotics but can also enhance the immune responses to eliminate the active *M. tb* infection.

## Data Availability Statement

The datasets generated for this study are available on request to the corresponding author.

## Ethics Statement

The studies involving human participants were reviewed and approved by Institutional Review Board of Western University of Health Sciences. The patients/participants provided their written informed consent to participate in this study.

## Author Contributions

AA synthesized cyclic peptide. RK and VV conceived the study, developed study design, analyzed the data, and prepared the manuscript. JH, DA, and RC conducted the studies and drafted the manuscript. AY, KT, RA, and TN provided technical assistance. All authors contributed to the article and approved the submitted version.

## Conflict of Interest

The authors declare that the research was conducted in the absence of any commercial or financial relationships that could be construed as a potential conflict of interest.
